# First person – Jaeike Faber

**DOI:** 10.1242/bio.058592

**Published:** 2021-02-15

## Abstract

First Person is a series of interviews with the first authors of a selection of papers published in Biology Open, helping early-career researchers promote themselves alongside their papers. Jaeike Faber is first author on ‘Quantified growth of the human embryonic heart’, published in BiO. Jaeike is a PhD student in the lab of Vincent Christoffels in the Department of Medical Biology and Cardiovascular Sciences, Amsterdam UMC, University of Amsterdam, The Netherlands, investigating evolution and development.


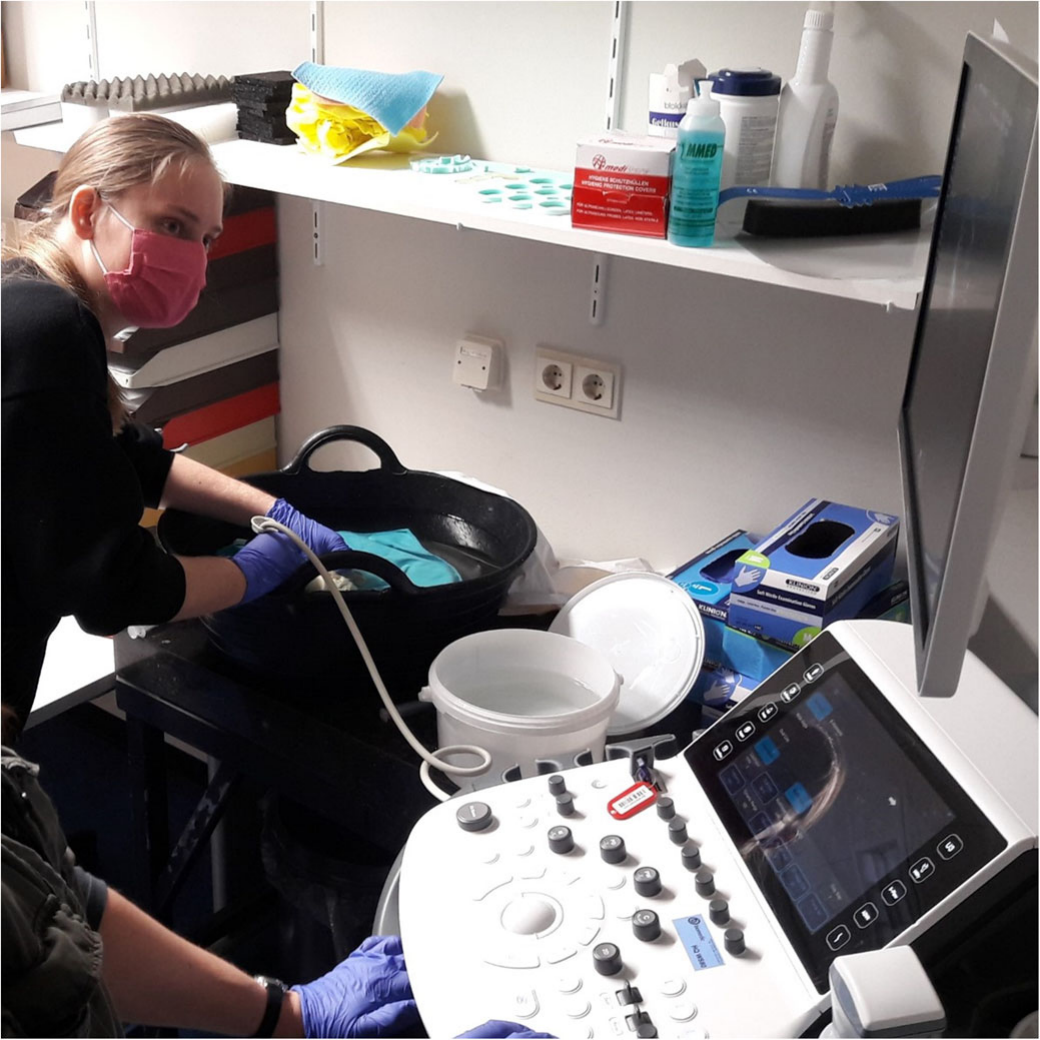


**Jaeike Faber**

**What is your scientific background and the general focus of your lab?**

I started studying biomedical sciences in 2009, the master I finished cum laude in 2017. In between, I also graduated as a medical doctor and did part of the masters in biology. Because of my interest in language evolution and phonology/phonetics, I additionally attended several linguistics courses. All this was at Leiden University in The Netherlands.

Most of my bio(medical) research has focused on heart development: from internships on human induced pluripotent stem-cell derived cardiomyocytes under supervision of Milena Bellin in the lab of Christine Mummery, to axial heart level in snakes in the group of Michael Richardson and Robert Poelmann. An exception was the work I did as a masters’ student in the group of Jakob Vinther at the Palaeobiology Research Institute in Bristol. There, I worked on scanning electron microscopy of the pigmented melanosomes isolated from various vertebrate tissues.

Currently, I am wrapping up my PhD on embryonic heart development in Vincent Christoffels’ lab. This lab strives to understand how congenital heart defects and cardiac arrhythmias develop, and how this is influenced by individual variations in genome function.

“This lab strives to understand how congenital heart defects and cardiac arrhythmias develop…”

**How would you explain the main findings of your paper to non-scientific family and friends?**

In our paper, the growth of the various cardiac components of the normal human heart during the first 8 weeks of development is quantified. Overall, the heart grows exponentially but some parts grow faster than others which is one of the processes by which the heart changes shape. The embryonic heart transforms from a smooth linear heart tube to a septated, four chambered heart. Additionally, in the paper's supplement, you can find interactive 3D models of the embryonic hearts from which the measurements were derived.

“ … in the paper's supplement, you can find interactive 3D models of the embryonic hearts …”

**What are the potential implications of these results for your field of research?**

Most congenital cardiac malformations are diagnosed by a first-trimester ultrasound. Techniques are advancing fast, which makes that malformations can be detected ever earlier in development. Our paper provides a reference work for embryonic cardiac growth.

In future, it might be possible that the cardiac growth of an embryo can be monitored. When this growth is then compared to a standardized growth curve, upcoming malformations could be diagnosed earlier since most congenital cardiac malformations are due to abnormal differential growth.

**What has surprised you the most while conducting your research?**

It surprised me most that the left atrium appears to develop its trabecular structure (pectinated musculature) later than the right atrium. It would be interesting to know how this process is regulated and if this delay in trabeculation serves a purpose.

**Figure:**
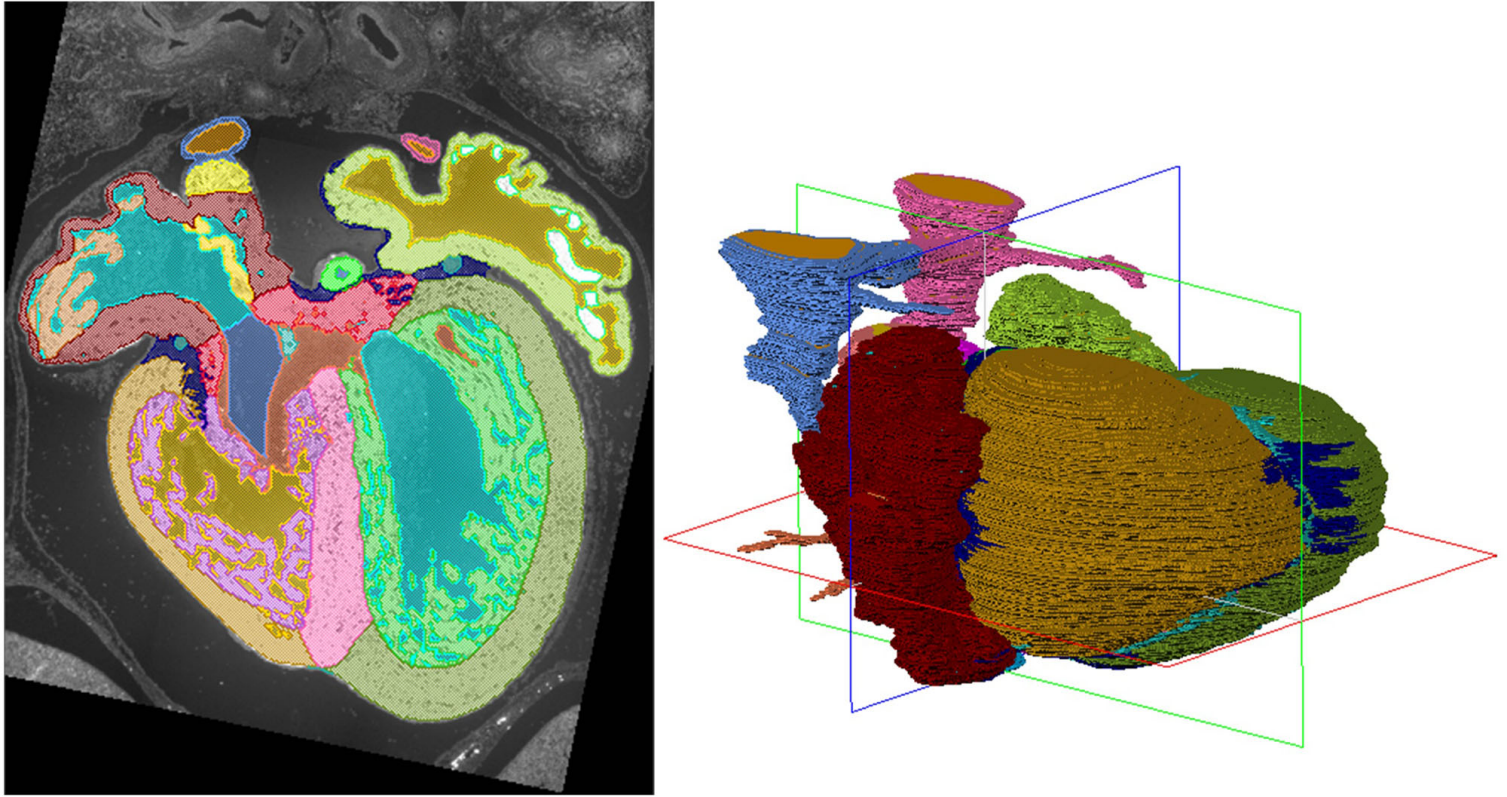
**Making an ultrasound of a preserved human foetal heart with tricuspid atresia.**

**What, in your opinion, are some of the greatest achievements in your field and how has this influenced your research?**

I admire the work done by the ‘old’ anatomists. By just using their eyes they have discovered so many things. For example, the works by Keith and Flack from 1907 and Benninghoff, 1933 were so comprehensive that they could still help me understand the development of the sinus venarum.

The old publications on heart anatomy of rarely studied animals, such as the one by Rowlatt, 1980, are also treasure troves of data. If we want to understand why a human heart develops, looks, and behaves the way it does, it helps to compare it to the hearts of other species. Preferably not only mouse and chicken, but also other mammals, birds and ectotherms. Luckily, this is something my co-promotor Bjarke Jensen is very familiar with!

**What changes do you think could improve the professional lives of early-career scientists?**

If society wants to stimulate good science, and would like to stimulate early-career scientists, it would be best if obtaining funding to do research would be made easier. In academia there is, as always, a shortage of funding, which makes the competition for it never-ending. Since writing catchy research proposals has nothing to do with doing good science, I think a lot of early-career scientists and good science are lost because of funding related issues.

**What's next for you?**

I hope to finish my PhD this year. Thereafter, I will start my residency in clinical pathology at the Erasmus MC in Rotterdam.
